# Development and Validation of a HTS Platform for the Discovery of New Antifungal Agents against Four Relevant Fungal Phytopathogens

**DOI:** 10.3390/jof9090883

**Published:** 2023-08-28

**Authors:** Rachel Serrano, Víctor González-Menéndez, José R. Tormo, Olga Genilloud

**Affiliations:** Fundación MEDINA, Av. Conocimiento 34, Health Sciences Technology Park, 18016 Granada, Spain; ruben.tormo@medinaandalucia.es (J.R.T.); olga.genilloud@medinaandalucia.es (O.G.)

**Keywords:** phytopathogenic fungi, fungicides, high-throughput screening, biocontrol agents

## Abstract

Fungal phytopathogens are the major agents responsible for causing severe damage to and losses in agricultural crops worldwide. *Botrytis cinerea*, *Colletotrichum acutatum*, *Fusarium proliferatum*, and *Magnaporthe grisea* are included in the top ten fungal phytopathogens that impose important plant diseases on a broad range of crops. Microbial natural products can be an attractive alternative for the biological control of phytopathogens. The objective of this work was to develop and validate a High-throughput Screening (HTS) platform to evaluate the antifungal potential of chemicals and natural products against these four important plant pathogens. Several experiments were performed to establish the optimal assay conditions that provide the best reproducibility and robustness. For this purpose, we have evaluated two media formulations (SDB and RPMI-1640), several inoculum concentrations (1 × 10^6^, 5 × 10^5^ and 5 × 10^6^ conidia/mL), the germination curves for each strain, each strain’s tolerance to dimethyl sulfoxide (DMSO), and the Dose Response Curves (DRC) of the antifungal control (Amphotericin B). The assays were performed in 96-well plate format, where absorbance at 620 nm was measured before and after incubation to evaluate growth inhibition, and fluorescence intensity at 570 nm excitation and 615 nm emission was monitored after resazurin addition for cell viability evaluation. Quality control parameters (RZ’ Factors and Signal to Background (S/B) ratios) were determined for each assay batch. The assay conditions were finally validated by titrating 40 known relevant antifungal agents and testing 2400 microbial natural product extracts from the MEDINA Library through both HTS agar-based and HTS microdilution-based set-ups on the four phytopathogens.

## 1. Introduction

Plant pathogens are one of the major problems affecting agriculture worldwide. They are responsible for the loss of one third of the crops produced annually [1,2]. Fungal phytopathogens are widely distributed and can impose plant diseases on a large variety of plants, including cereals, vegetables, and fruit. They can infect plants through various mechanisms of pathogenesis, often accompanied by the biosynthesis of mycotoxins, which generates important yield losses that have a considerable economic impact on the agricultural sector [3].

Based on their scientific/economic importance, the top ten fungal phytopathogens have been identified [4] in rank order as (i) *Magnaporthe oryzae*, a phytopathogen model for plant host studies that, together with *M. grisea*, is one of the main causal agents of blast rice, the most destructive plant disease [5,6,7]; (ii) *Botrytis cinerea*, responsible for gray mold, which generates severe damages and losses in fruit, vegetables, and ornamental plants during both pre- and post-harvesting steps [4,8,9,10]; (iii) *Puccinia* spp., the cause of three types of royal diseases in *Triticum aestivum* which seriously impact wheat production, mainly via the infection of *P. graminis*, *P. striiformis*, and *P. triticina* [4]; the genus *Fusarium*, which includes a high number of pathogenic species, can produce numerous mycotoxins and colonize plants in a wide range of climatic areas; (iv) *Fusarium graminearum*, (v) *Fusarium oxysporum*, and *Fusarium proliferatum*, which can infect a broad range of crops such as maize, wheat, rice, soybean, barley, fruit, and even ornamental plants, where one of the most significant damages is root rot [11]; (vi) *Blumeria graminis*, which is the causal agent of powdery mildew in barley and wheat crops and is considered a high-risk pathogen because of its potential to change its virulence [12]; (vii) *Zymoseptoria tritici*, which is responsible for the most important wheat disease, the Septoria leave blotch, with a high percentage of infection, inducing significant damages; (viii) *Colletotrichum* spp., which are the genera that promote the anthracnose disease in more than 3000 plant species and can alternate between different infection stages (hemibiotrophic, necrotrophic, and biotrophic), making it very efficient in propagation and leading to considerable worldwide economic losses in fruit and vegetable crops [4,13,14,15,16]; (ix) *Ustilago maydis*, a homobasidiomycete that normally infects maize (*Zea mays*) through a complex pathogenic process, including morphogenetic transitions until its sexual cycle is completed [17]; and finally, (x) *Melampsora lini*, responsible for the rust disease on *Linum usitatissimum*, which causes severe losses in seed yields as well as a fiber quality reduction in flax plants [18].

Nowadays, the preferred strategy for managing plant diseases and crop protection is the use of a broad range of chemical agents such as fungicides combined with herbicides and insecticides. The use of synthetic fungicides has enabled the efficient control of fungal infections and improved the production yields and quality of crops [19,20,21]. However, the continuous and extended use of these agrochemicals has shown a severe negative impact on both environmental and human health [19,22]. Chemical fungicides accumulated in soils, plants, and water may seriously affect humans through the food chain [22], and their application has become strictly regulated to reduce their toxicity in agricultural fields and guarantee food safety. Given these current limitations, there is an urgent need to develop safe, efficient, and environmentally friendly next-generation fungicides.

The most common methods to identify antifungal agents against filamentous fungi include agar dilution and disc diffusion assays on Petri dishes [23,24]. These traditional methods are time-consuming, expensive, and require large quantities of material to be tested [23]. The introduction of a miniaturized High-throughput Screening (HTS) platform for agar-based assays by our group fixed some of these problems [25]. However, to date, few methods for the determination of the susceptibility of phytopathogens to fungicides in in vitro set-ups using liquid conditions have been reported. Specifically, the microdilution methodology provides the advantages of simplicity, reliability, and time- and cost-effectiveness and could be scalable for a large number of samples, which makes it ideal for wide screening campaigns looking for new fungicide agents [23,24].

Natural microbial products are an untapped source of new bioactive molecules with broad applications. The use of natural agents for the biocontrol of phytopathogens offers an attractive alternative to enable economical and environmentally sustainable agriculture in the forthcoming decades [26,27,28]. HTS approaches with natural products allow the for both the evaluation of pure compounds and the extraction of mixtures for the rapid identification of new potential antifungal agents with a simple and accurate detection technique in a miniaturized assay with reduced costs.

Therefore, in this study, we have developed two miniaturized HTS methodologies based on agar and liquid assays to identify and characterize the antifungal properties of a wide variety of samples (pure compounds and natural product mixtures) against four relevant fungal phytopathogens: *Botrytis cinerea*, *Colletotrichum acutatum*, *Fusarium proliferatum*, and *Magnaporthe grisea*. In this sense, the final objective of this study was to provide a validated HTS platform to enable the efficient discovery of next-generation fungicides for the biocontrol of these four important phytopathogens.

## 2. Materials and Methods

### 2.1. Fungal Strains and Conidia Production

Four fungal strains were obtained from CBS-KNAW Fungal Biodiversity Centre, CECT Spanish Type Culture Collection and MEDINA Fungal Collection. *Botrytis cinerea* B05.10 (CECT 20754) was isolated from *Vitis vinifera* in Italy, *Colletotrichum acutatum* CF-137177 was isolated from a dead leaf collected in Mexico, *Fusarium proliferatum* CBS 115.97 was isolated from *Dianthus caryophyllus* in Italy, and *Magnaporthe grisea* CF-105765 was isolated from the ear blast on *Eleusine indica* collected in Argentina.

Each phytopathogen was cultured in five solid media and five liquid media to optimize their sporulation process. The solid media evaluated were 2% malt agar (MEA), corn meal agar (CMA), oatmeal agar (OAT), potato dextrose agar (PDA), and yeast-malt agar (YM) [25]. The submerged culture conditions assessed were also achieved by using three traditional media following a previously described protocol (SDB, PDB, and SMY) [25,29,30] and two new formulations: GMS medium (glycerol 5 g/L, Bacto malt extract 10 g/L, Difco soy peptone 10 g/L, Difco dextrose 4 g/L, ammonium sulfate 2 g/L, KH_2_PO_4_ 1 g/L, MgSO_4_·7H_2_O 0.5 g/L, FeSO_4_·7H_2_O 0.005g/L, pH adjusted at 7) and VME medium (vegetable peptone Biochemika 10 g/L, Bacto malt extract 40 g/L, Difco yeast extract 0.4 g/L, (NH_4_)2SO_4_ 2.4 g/L, pH adjusted at 6.6).

Conidia solutions were obtained following different protocols depending on the sporulation condition (solid or liquid). After 14–28 days of incubation (specific timing for each strain, the surfaces of the sporulated agar plates (*n* = 5) were scraped with a sterile loop and a Tween 80 solution 0.1% (*v/v*), and the resulting suspension was filtered through sterile cotton gauzes. In the case of cultures in liquid conditions, each inoculum tube containing 16 mL of media was directly filtered and washed to remove medium remains and therefore obtain a final homogeneous solution. The resulting conidia stocks were adjusted to a final volume of 10 mL, and the concentration of conidia per milliliter was determined by counting in a Neubauer chamber 0.0025 mm^2^. All experiments were performed on three different days/batches to compare the robustness of production and select the optimal condition and method for each fungal strain.

### 2.2. Agar-Based Assays against Fungal Phytopathogens

Agar-based assays against *C. acutatum*, *F. proliferatum*, and *M. grisea* have been already described by our group [25], where the optimal conditions were successfully determined to allow for the evaluation of the antifungal activity of microbial extracts. However, the agar-based assay of *B. cinerea* required new adjustments to be made to the different parameters. Following the steps described for the other three phytopathogens [25], *B. cinerea* conidia were inoculated in Sabouraud Dextrose Agar medium (SDA) adjusted in a range from 1 × 10^4^ to 5 × 10^7^ conidia/mL. Once solidified, the samples were dispensed on the top of the agar containing the inoculum. Five antifungal compounds were evaluated as positive controls to determine the optimal concentrations for a reference Dose Response Curve (DRC): amphotericin B (AmB), azoxystrobin, cycloheximide, cyproconazole, and econazole. The plates were incubated at 25 °C and 70% relative humidity (RH) at different times, with 48 h proving to be the optimum time. The diameter and turbidity of the inhibition halos were calculated using proprietary Image Analyzer^®^ software (version 1.0.3393.20294) and a stereoscope (Leica™ MZ16).

### 2.3. Liquid-Based Assays against Fungal Phytopathogens

The methodology developed in this work was adapted from the previously described HTS protocol for the human fungal pathogen *Aspergillus fumigatus* [31]. Two media formulations were compared to select the assay medium: Sabouraud Dextrose Broth (SDB) was the reference medium for the agar-based assay and is commonly used in mycology laboratories [25,32,33], and RPMI-1640 was already used for the *A. fumigatus* antifungal assays and is also recommended by the CLSI (Clinical & Laboratory Standards Institute) and the EUCAST (European Committee on Antimicrobial Susceptibility Testing) [31]. Several experiments were performed to establish the optimal inoculum concentration which provided the highest reproducibility and robustness for each phytopathogen: 1 × 10^6^ conidia/mL (same used in the agar-based assay), 5 × 10^5^, and 5 × 10^6^ conidia/mL. Plates were incubated at 25 °C and 70% RH from 8 to 168 h to define the growth curve for each phytopathogen and determine the optimal incubation times. Absorbance at 620 nm was measured in a spectrofluorometer ENVISION™ Multilabel Reader (PerkinElmer, Waltham, MA, USA) before and after incubation. Finally, for the cell viability evaluation, 10 µL of resazurin at 0.02% (*w/v*) was added, and fluorescence intensity at 570 nm excitation and 615 nm emission was monitored from 5 min to 4 h of incubation at 37 °C for dye reduction evaluation [23,31,32].

### 2.4. Validation of Assay Conditions

Once the optimal assay conditions were established for each plant pathogen, primary screening with 40 known antifungal standards was performed to validate the assay parameters. The samples were tested in triplicate, and active compounds were evaluated in DRC. Finally, the assays were also validated with a subset of 2400 microbial extracts (natural products mixtures). For this purpose, extracts were selected from the MEDINA microbial extract Library, obtained from microbial fermentations of both actinomycetes and fungi.

The assays were performed in Corning^®^ Costar™ 96-well plates containing a set of control wells in the first and last columns according to the following layout: the four top wells of the first column (1A–1D wells) with the positive control (inoculum + AmB in DMSO), the four bottom wells (1E–1H) with the negative control (inoculum + 2% DMSO), and the last column (A12–H12) containing a reference DRC of AmB in 2% DMSO. The samples were first dispensed (2–10 µL) in columns 2–11 with an automated liquid handler Biomek i7, Beckman Coulter^®^ (Life Sciences Division Headquarters, Indianapolis, IN, USA), then the inoculum was added to a final volume of 100 µL using a Thermo Scientific Multidrop Combi (MTX Lab System, Vienna, VA, USA).

### 2.5. Data Analysis

Absorbance and fluorescence raw data were used to calculate antifungal activity via the Genedata Screener^®^ software (Genedata AG, Basel, Switzerland). Absorbance values for each well were obtained from the difference between the absorbance measured before and after incubation time. The percentage of inhibition (% INH) was determined for each sample by the following equations integrated in the software. For the absorbance-based assay, % INH = (Abs_test_ − Abs_pos_)/(Abs_neg_ − Abs_pos_), where Abs_test_ was the absorbance of the evaluated sample, and Abs_pos_ and Abs_neg_ were the absorbances for the positive and negative controls. For the fluorescence-based assay, % INH = 100−% Reduction and % Reduction = 100 × (FI_test_ − FI_pos_)/(FI_neg_ − FI_pos_), where FI_test_, FI_pos_, and FI_neg_ corresponded to the fluorescence intensity of a given evaluated sample and positive and negative controls, respectively.

DRCs of the studied standards were calculated following a sigmoidal shape and adjusted to a classical hill equation integrated in the software: E/Emax = A^n^/(IC_50_^n^ + A^n^), where E corresponded with the response, A was the compound concentration, IC_50_ was the compound concentration that produced 50% of the maximal response, and n was the Hill coefficient, which was calculated using the following formula: n = log(81)/log(EC_90_/EC_10_), with EC_90_ and EC_10_ being the concentrations that produced 90% and 10% of the maximal response, respectively. The quality control (QC) parameters—Robust Z Factor (RZ’ Factor), Signal/Background ratio (S/B), and the IC_50_ (µg/mL) of the positive control AmB—were determined for each batch of assay plates.

Additional statistical analysis using JMP^®^ software was applied to distribute and categorize the activity population obtained from the piloting HTS approach.

## 3. Results and Discussion

### 3.1. Induction of the Sporulation of Fungal Phytopathogens

The first step of the HTS assay development involved the definition of a standardized method for conidia production. The optimal condition to induce the sporulation was specific for each of the four phytopathogens tested (Table 1). *B. cinerea* only produced a sufficient concentration of conidia for the assay on solid media, specifically when it was cultured on OAT medium for 21 days at 25 °C (where the concentration obtained was 3.7 × 10^7^ ± 1.9 conidia/mL). The other three strains produced conidia in both solid and liquid culture conditions (Table 1). In the case of *C. acutatum*, no significant differences were observed in the concentration of conidia: 8.8 × 10^7^ ± 1.7 conidia/mL from solid culture on YM medium after 21 days, and 7.8 × 10^7^ ± 1.9 conidia/mL from submerged culture in SMY liquid medium (220 rpm) for 3 days. For *F. proliferatum*, the best sporulation was obtained from the overnight inoculum in SMY medium (2.5 × 10^8^ ± 0.4 conidia/mL), in agreement with previous published data [25]. Finally, *M. grisea* produced the highest concentration of conidia on CMA agar plates (2.7 × 10^8^ ± 1.0 conidia/mL), although no significant differences were observed in liquid conditions when cultured for 2 days in the new medium formulation VME (2.3 × 10^8^ ± 0.9 conidia/mL).

In general, the best conditions for the sporulation of *B. cinerea* and *F. proliferatum* were cultivation in OAT agar plates and in the SMY submerged tube, respectively. However, for *C. acutatum* and *M. grisea*, both solid and liquid cultures provided similar conidia production rates. Significant advantages in time and cost requirements were clearly observed for liquid cultivation when compared to solid conditions (Table 2), and therefore, the liquid culture in SMY was selected for the sporulation of *C. acutatum*, and VME was selected for *M. grisea*.

### 3.2. Botrytis cinerea Agar-Based Assay

**Figure 1:**
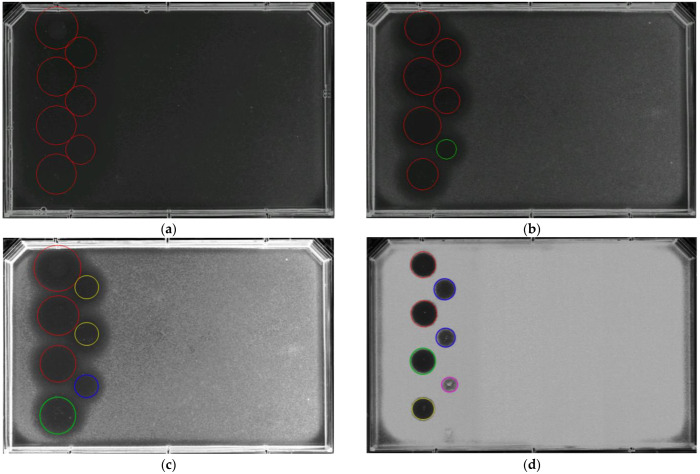
Agar-based assay using four different concentrations of *Botrytis cinerea* conidia: (**a**) 1 × 10^5^ conidia/mL, (**b**) 5 × 10^5^ conidia/mL, (**c**) 1 × 10^6^ conidia/mL, and (**d**) 5 × 10^6^ conidia/mL. Inhibition zones corresponded to the AmB curve starting at 200 µg/mL with eight-point 1:2 serial dilutions to 1.56 µg/mL. Inhibition halos were highlighted based on diameter (cm) and turbidity (circle color) to assess the antifungal activity of the compounds. The halos were sorted into five categories depending on the conidia germination rates: halo A (red) corresponds to the highest activity where germination was not observed, halo B (green) where 25% of conidia started to germinate, halo C (yellow) for 50% of germination, halo D (blue) for 75% of germination, and halo E (pink) for 100% germination with high turbidity, indicating the lowest activity.

Four final concentrations of *B. cinerea* conidia in SDA medium were compared: 1 × 10^5^, 5 × 10^5^, 1 × 10^6^, and 5 × 10^6^ conidia/mL (Figure 1) [25,34]. Concentrations of 1 × 10^5^ and 5 × 10^5^ conidia/mL generated a very sparse and translucent layer of mycelium growth that required long incubation times to show good defined inhibition halos (Figure 1a,b). On the contrary, 5 × 10^6^ conidia/mL generated a very dense mycelium growth more resistant to AmB (Figure 1d). The concentration of 1 × 10^6^ conidia/mL showed the best definition of the inhibition zones for the AmB curve, providing a good assay window between positive and negative controls (Figure 1c). Therefore, the optimal concentration for the *B. cinerea* agar-based assay was considered to be 1 × 10^6^ conidia/mL. AmB at 100 µg/mL, and 20% DMSO were used as positive and negative controls, respectively. The AmB reference curve included 1:2 serial dilution concentrations from 50 µg/mL to 6.25 µg/mL. The plates were incubated for 48 h at 25 °C and 70% RH to ensure clear zones of inhibition.

### 3.3. Botrytis cinerea Microdilution Assay

**Figure 2:**
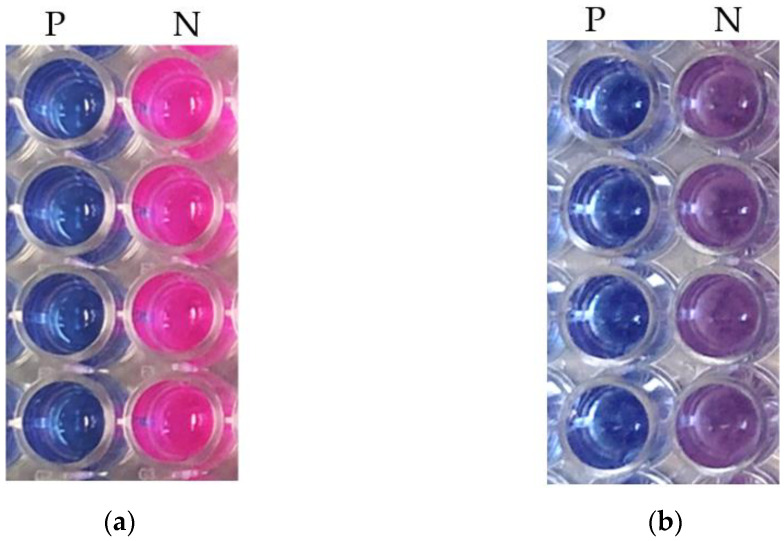
Resazurin reaction in the control wells in (**a**) RPMI-1640 medium and (**b**) SDB medium. **P** indicates the positive control wells (growth inhibition), whereas **N** indicates the negative control wells (normal growth).

The first step to develop the antifungal assay with *Botrytis cinerea* using the microdilution method was to determine the best liquid medium that allowed for optimal signal detection from the two different media under comparison: SDB and RPMI-1640 [25,31,32,33]. *B. cinerea* developed good growth in both media, with all conidia germinating and developing mycelium. The absorbance raw data was similar in both media, but the fluorescence readouts showed some differences that could be observed visually and when fluorescence intensity was measured. Positive control wells remained blue (indicating absence of metabolic activity and no germination), whereas negative control wells turned into a fluorescent pink color (indicating metabolic activity and conidia germination) [31]. When *B. cinerea* was grown in the RPMI medium, we observed that the described blue color in the inhibited wells (Figure 2aP) corresponded to a fluorescence intensity of 605 ± 10 RFU, and a fluorescent pink color in the negative control wells (Figure 2aN) corresponded to a higher fluorescence intensity of 13,450 ± 292 RFU. However, the viability assay in SDB medium showed a purple color in negative controls (Figure 2bN), with lower fluorescence values of 8063 ± 420 RFU. In addition, the two specific fungi media SMF1 [35] and PDB [30] were evaluated, showing similar results for the fluorescence readouts than those obtained for the SDB medium [30,35]. After statistical analysis, the S/B and RZ’ factors were determined (120 wells of negative controls and 120 wells of positive controls) and were significatively better in the RPMI medium, highlighting it as the optimal medium for signal detection.

**Figure 3:**
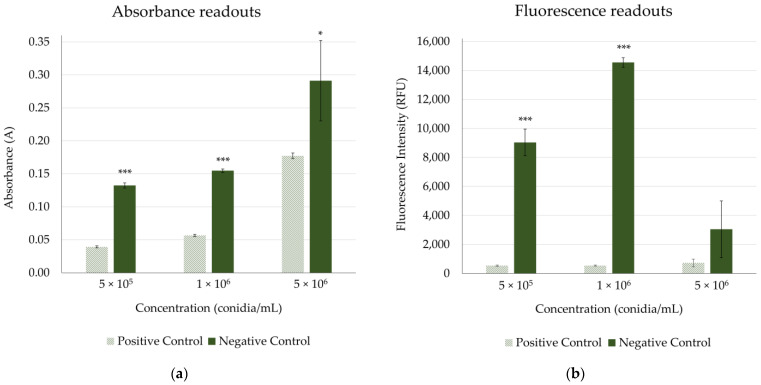
Influence of three concentrations of *Botrytis cinerea* conidia on (**a**) absorbance and (**b**) fluorescence readouts in RPMI-1640 medium. Positive control corresponds to AmB at 100 µg/mL in 2% DMSO, and negative control corresponds to 2% DMSO (final concentrations). * *p* < 0.05, *** *p* < 0.001.

The best three inoculum concentrations obtained from the *B. cinerea* agar-based assay were evaluated for the microdilution assay: 5 × 10^5^, 1 × 10^6^ and 5 × 10^6^ conidia/mL. The growth inhibition assay showed that 5 × 10^6^ conidia/mL generated very dispersed absorbance data with considerably low QC parameters (Table 3 and Figure 3). This could be due to the saturated background generated by the addition of a high concentration of *B. cinerea* conidia. Lower concentrations of 5 × 10^5^ and 1 × 10^6^ conidia/mL presented more homogeneous and robust absorbance data without significant differences between RZ’ factors (0.77 and 0.79, respectively) (Table 3). However, the viability assay clearly supported 1 × 10^6^ conidia/mL as the optimal concentration, with the highest RZ’ (0.89) and S/B (23.19), in agreement with Pelloux-Prayer et al. 1998, who reported this conidia concentration as the best one for a fluorescence test using Alamar blue [36]. Therefore, 1 × 10^6^ conidia/mL was selected as the optimal concentration that provided robust data with a broad assay window for absorbance and fluorescence readouts for the *B. cinerea* assay.

**Figure 4:**
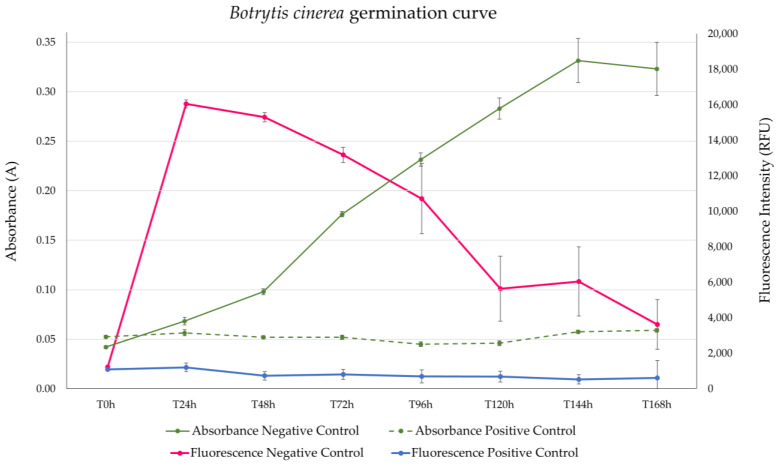
Germination curves of *Botrytis cinerea* conidia based on absorbance at 612 nm (green lines) and fluorescence at 570 nm excitation and 615 nm emission (pink and blue lines). The left axis quantifies the absorbance values of negative controls (green continued line) and positive controls (green discontinued line). The right axis measures the fluorescence intensities of negative controls (pink line) and positive controls (blue line). Each data point represents an average of 40 wells with their corresponding standard deviation.

The growth curve of *Botrytis cinerea* in the presence of positive and negative controls was analyzed to determine the optimal time for assay incubation. The absorbance values of the negative controls increased with time, while the positive controls remained constant (Figure 4, both green lines). Consequently, the S/B ratio also increased at longer incubation times after 48 h. The RZ’ factors showed robustness until 120 h of incubation (RZ’ > 0.7); hence, the optimal time for the absorbance readout was determined to be in the range of 48 to 120 h. On the other hand, the fluorescence readouts presented the highest assay window when resazurin was added after 24 h of incubation but decreased with longer incubation times as the fluorescence of negative controls could not reach the maximum fluorescence intensity. This effect could be related to the presence of a high metabolic activity due to the high amount of germinated conidia, which could rapidly reduce the resazurin to hydroresorufin (non-fluorescent) and generate a false signal of viability. The RZ’ factor also decreased with longer times, being acceptable until 72 h of incubation (RZ’ > 0.7). Then, the best readout time for the viability assay of *B. cinerea* (fluorescence) was defined to be in the range of 24 to 72 h, with incubation for 72 h allowing for the evaluation of both the growth inhibition and cell viability of *B. cinerea* conidia within the same plate set-up.

In order to evaluate the reproducibility of the method based on the QC parameters, the antifungal assay was performed on three different days using three independent batches of sporulated plates and fresh reagents. The growth inhibition assay showed absorbance values for *B. cinerea* similar for each replicate (0.23 ± 0.01 A), RZ’ factors higher than 0.70 (0.80 ± 0.05), and good S/B ratios (48.95 ± 10.10). Regarding the viability assay, it showed greater homogeneity with the fluorescence data of the negative controls (13,173 ± 427 RFU), with RZ’ factors close to 1.00 (0.94 ± 0.03) and S/B ratios (24.98 ± 5.00) slightly lower than absorbance readouts but with less variability. In general, these parameters indicated that the established assay conditions provided data that were robust enough to evaluate the antifungal activity of large sets of samples against *Botrytis cinerea* in HTS format.

**Figure 5:**
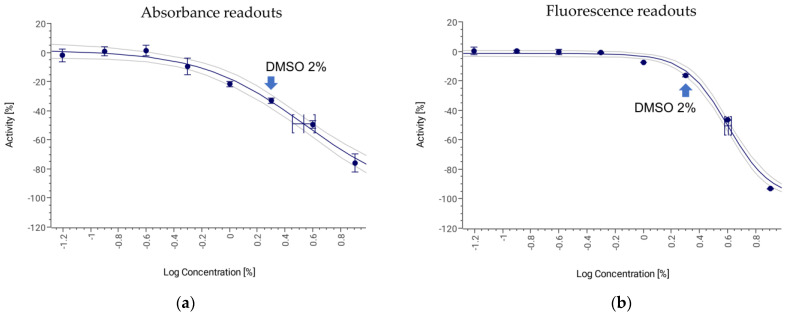
Tolerance of *Botrytis cinerea* conidia to different DMSO concentrations (**a**) based on absorbance readouts (R^2^ = 0.97) and (**b**) fluorescence readouts (R^2^ = 0.99).

The tolerance of *B. cinerea* to dimethyl sulfoxide (DMSO), the most common solvent used in compound libraries, was determined to evaluate its possible effect on conidia germination. Figure 5 shows the germination inhibition in a 1:2 serial dilution concentration-dependent curve starting at 8% DMSO. A final concentration of 4% DMSO still induced significant effects on fungal growth (Figure 5a) and on the viability of *B. cinerea* conidia (Figure 5b), where the IC_50_ was determined to be between 3.43 (absorbance) and 4.01% (fluorescence). *B. cinerea* germination exhibited a clear decrease when conidia were exposed to concentrations higher than 2%. These results showed that natural product extracts and compound libraries prepared in DMSO can be tested using this methodology if the final assay concentration of this solvent remains below 2%.

**Figure 6:**
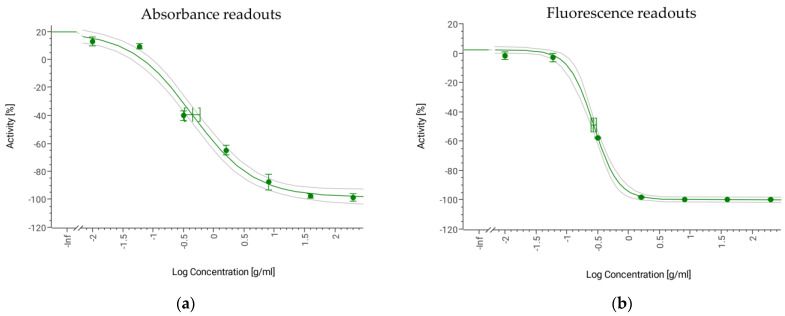
AmB DRC against *Botrytis cinerea* based on (**a**) absorbance readout IC_50_ = 0.45 µg/mL (0.33–0.60 µg/mL) (R^2^ = 0.98) and (**b**) fluorescence readout IC_50_ = 0.27 µg/mL (0.25–0.30 µg/mL) (R^2^ = 0.99).

The broad spectrum antifungal agent Amphotericin B (AmB) was tested as the positive control. This natural polyene inhibits human and plant pathogens by binding with ergosterol [37,38,39,40,41]. Different stock concentrations of AmB dissolved in 100% DMSO were tested in a DRC to determine the IC_50_ (µg/mL) and the sensitivity of the assay. The AmB DRC started at a final concentration of 200 µg/mL with eight-point 1:5 serial dilutions to 2.56 ng/mL. The IC_50_ of AmB for *B. cinerea* for absorbance readouts was 0.45 µg/mL (0.33–0.60 µg/mL), and for the fluorescence intensity, the IC_50_ was 0.27 µg/mL (0.25–0.30 µg/mL) (Figure 6a,b).

### 3.4. Colletotrichum acutatum Microdilution Assay

The same steps and QC parameters were evaluated for the development of the *C. acutatum* antifungal assay. Two concentrations of conidia (5 × 10^5^ and 1 × 10^6^ conidia/mL) were evaluated to select the optimal inoculum concentration based on the results obtained for *B. cinerea*. Although no significant differences were observed between the assay window at both concentrations (S/B was 47.18 and 55.12 for absorbance and 15.68 and 18.76 for fluorescence, respectively), the RZ’ factors were slightly higher with 1 × 10^6^ conidia/mL (RZ’ = 0.76), and this concentration was selected for the *C. acutatum* assay. The germination of *C. acutatum* in RPMI medium was monitored at 8 h intervals given the rapid germination and growth of this pathogen. After 24 h of incubation, the RZ’ factors started to increase (>0.5), but the S/B was still low (11.70). The optimal incubation time for the *C. acutatum* assay plates was 40 h with the following RZ’ and S/B values: absorbance readouts RZ’ = 0.75 ± 0.01 and S/B = 53.05 ± 5.50 and fluorescence readouts RZ’ = 0.91 ± 0.01 and S/B = 17.01 ± 1.35. The DMSO-tolerance evaluation revealed that this strain remained unaffected up to 2% DMSO and exhibited a significant decrease in growth when it was exposed to concentrations higher than 4%. The AmB DRC with eight-point 1:2 serial dilutions for *C. acutatum* starting at 10 µg/mL final concentration enabled the determination of an IC_50_ of 1.25 µg/mL (1.12–1.33 µg/mL) for the absorbance readouts and 1.21 µg/mL (1.19–1.23 µg/mL) for the fluorescence readouts (Table 4).

### 3.5. Fusarium proliferatum Microdilution Assay

The antifungal assay against *F. proliferatum* was also developed following the same steps. For the absorbance-based assay, no significant differences were observed in the RZ’ value using both conidia concentrations (5 × 10^5^ and 1 × 10^6^ conidia/mL) (0.72 for both). However, 1 × 10^6^ conidia/mL provided a clear increase in the S/B values (from 14.49 to 59.06). The optimal time for the incubation of assay plates was 20 h due to the faster growth rate of this strain, with RZ’ = 0.77 ± 0.05 and S/B = 57.71 ± 7.04. After 2 h of incubation with resazurin, the fluorescence readouts provided the most robust values, where the QC parameters were RZ’ = 0.94 ± 0.01 and S/B = 17.54 ± 1.76. Tolerance to DMSO by *F. proliferatum* showed a normal rate of conidia germination up to 4% DMSO. *F. proliferatum* is the most resistant strain of the panel to AmB. IC_50_s were determined for both readouts in a DRC starting at 100 µg/mL with eight-point 1:2 serial dilutions to 0.78 µg/mL; the IC_50_s for absorbance and fluorescence were 6.77 µg/mL (6.11–7.51 µg/mL) and 6.75 µg/mL (6.41–7.12 µg/mL), respectively (Table 4).

### 3.6. Magnaporthe grisea Microdilution Assay

No significant differences were observed in the RZ’ and S/B values when two concentrations of conidia/mL were compared in the development of the *M. grisea* antifungal assay. The main difference was the time required to complete the resazurin reduction process (more than 3 h for 5 × 10^5^ conidia/mL). Therefore, 1 × 10^6^ conidia/mL was selected as the concentration for the *M. grisea* inoculum. In this set-up, the assay plates were incubated for 48 h to ensure higher QC parameters for the absorbance readouts (RZ’ = 0.81 ± 0.03 and S/B = 54.21 ± 5.89) and for 1 h and 30 min with resazurin for the fluorescence-based viability assay (RZ’= 0.95 ± 0.01 and S/B = 13.62 ± 1.65). DMSO-tolerance curves showed that *M. grisea* can accept up to 2% DMSO final concentration before conidia germination is affected. The best DRC for the standard AmB started at a final concentration of 50 µg/mL with eight-point 1:2 serial dilutions to 0.39 µg/mL; the IC_50_ = 4.51 µg/mL (4.33–4.69 µg/mL) for the absorbance readouts and 5.38 µg/mL (5.30–5.46 µg/mL) for the fluorescence readouts (Table 4).

### 3.7. Sensitivity and Resistance to Antifungal Standards

The antifungal potential of 40 known fungicide agents was evaluated using both agar and microdilution methods (Table 5). Compound stocks were prepared at 1 mg/mL in 100% DMSO, from which 2 µL was diluted to a final assay volume of 100 µL to avoid the inhibitory effects of the solvent. The four phytopathogens were resistant to benalaxyl, cytochalasin, dimethomorph, ethaboxam, flutianil, iprovalicarb, mandipropamid, metalaxyl, radicicol, and validamycin at the maximum assay concentrations. The remaining 30 standards with inhibitory effects against one or more phytopathogens of the panel were then titrated in DRC using both methodologies (agar-based and microdilution assays). The standard curves of the compounds started at 20 µg/mL (final assay concentration) with 10-point 1:2 serial dilutions to 0.04 µg/mL. The Minimal Inhibitory Concentration (MIC) for the agar-based assays was determined to be the lowest concentration that generated an inhibition zone (halo diameter > 5 mm), and IC_50_ was determined to be the concentration responsible for 50% of the inhibition in the microdilution assays. Both values were used to confirm the antifungal activity of the different chemical classes of the compounds and were compared with previously described data (Table 5).

Azoxystrobin, benomyl, cycloheximide, cyprodinil, difeconazole, ferbam, fludioxonil, mandestrobin, mefentrifluconazole, metconazole, pydiflumetofen, pyraclostrobin, thiabendazole, trifloxystrobin, and triflumizole presented a broad antifungal spectrum against the four phytopathogens. In general, strobirulins (azoxystrobin, mandestrobin, pyraclostrobin, and trifloxystrobin) were the most effective agents. This group of compounds are inhibitors of cellular respiration through the complex III *cytochrome bc1* (ubiquinone oxidase) Qo site [42]. They have an extended antifungal spectrum on many filamentous fungi and are considered the most important agricultural fungicide group. Within this chemical class, pyraclostrobin was the most active compound among the standards, with MIC and IC_50_ values consistently low for the four phytopathogens (Table 5), in agreement with previous reports [43,44].

The most relevant chemical classes in use were triazoles, imidazoles, and benzimidazoles, which were effective against more than one plant pathogen. The most active triazoles were metconazole, difeconazole, and mefentrifluoconazole, inhibiting the four strains. Metconazole required the lowest concentration to inhibit fungal development in both assay methods (agar and microdilution), mainly against *F. proliferatum* (MIC 31.25 µg/mL and IC_50_ < 0.04 µg/mL), confirming its reported potential to control the Fusarium head blight of wheat (FHB) [45,46]. The remaining five triazoles provided broad activity profiles with some specificities between the two assay methods. Cyproconazole, flutriafol, and tetraconazole were totally inactive against *M. grisea*, while fenbuconazole and itraconazole only showed very poor inhibitory effects against *F. proliferatum*, suggesting that higher concentrations of the compounds may be required to reach the MIC and IC_50_ values.

Both imidazoles (triflumizole and fenamidone) presented two different activity patterns. Triflumizole was more effective, with significatively lower MIC and IC_50_ values for the four phytopathogens (in line with the broad antifungal spectrum reported in the literature and its effects on mycelial growth, spore germination, and germ tube elongation) [47]. Fenamidone, a very effective fungicide for *Oomycete* diseases such as downy mildew and certain leaf spot diseases [48], also showed inhibitory effects against *C. acutatum* and *M. grisea*. Finally, the benzimidazoles thiabendazol and benomyl, which affect the polymerization of β-tubulin and have been reported as fungicides for the control of the *Fusarium* species of a variety of crops [49], also exhibited inhibitory activity on other species, such as *B. cinerea*, *C. acutatum*, and *M. grisea*.

Other interesting antifungal agents tested were those that affect the synthesis of proteins. Among them, cycloheximide was the most active one, exhibiting inhibition of the four phytopathogens with both assay methodologies, which confirmed its previously reported antifungal potential [50]. Cyprodinil showed higher inhibitory activity against *B. cinerea* in particular, with MIC (7.81 µg/mL) and IC_50_ values (0.06 µg/mL for the absorbance readouts and 0.29 µg/mL for the fluorescence readouts) that were significatively lower than those obtained for the other pathogens. This higher potency might be explained by cyprodinil’s mode of action, as it inhibits the biosynthesis of methionine, which affects the secretion of hydrolytic enzymes and is related to the mechanism of pathogenesis of *B. cinerea* [51]. Finally, in this group, the nucleosidic natural products tunicamycin and tubercidin, produced by *Streptomyces* species and previously reported to have antifungal activity against various human and plant pathogens [52,53], showed high specificity against *B. cinerea* and *C. acutatum*, an activity not reported to date.

Inhibitors of cellular respiration through the complex II succinate dehydrogenase (SDHI) were very effective with respect to controlling the *B. cinerea* strain. Within this group, pydiflumetofen was the most effective at inhibiting the four phytopathogens in both methodologies, well in line with previous reports of its broad antifungal spectrum [54,55]. Fluopyram and isofetamid showed a more specific activity profile, being fluopyram more active against *F. proliferatum* and isofetamid more active against *M. grisea*. Boscalid was highly and specifically active against the germination and viability of *B. cinerea*, with IC_50_ values of 0.33 µg/mL (absorbance) and 0.70 µg/mL (fluorescence), in agreement with previous reported data in growth inhibition tests (0.7 µg/mL to inhibit 50% of mycelial growth) [56,57]. *C. acutatum* resistance to this chemical class can be explained by previous reports of a natural resistance in different *Colletotrichum* species to SDHI fungicides [58,59].

Fludioxonil and cyclosporin A, with similar modes of action affecting signal transduction, have also been described for the control of different pathogens. Fludioxonil confirmed its broad antifungal spectrum, being more effective against *B. cinerea*. Several in vitro and field studies have determined this compound to be one of the most effective fungicides for the control of this phytopathogen through the inhibition of mycelial growth, germination, and conidiation [57]. Cyclosporin A (previously reported to have antifungal properties against *Candida* species) also showed inhibitory effects on *B. cinerea* and weak activity against *C. acutatum* [60,61], activities that have never been reported.

The last four antifungal standards belong to diverse chemical classes and present different modes of action. The picolinamide class is represented by fenpicoxamid, a semisynthetic modification of the natural compound UK-2A [62,63]. This fungicide, mainly used in cereals for the control of *Z. tritici*, was also active against *B. cinerea* and *M. grisea*. The dithio-carbamate ferbam, which has been described as a multi-site contact inhibitor for several phytopathogens [42,64,65], demonstrated activity against the four tested strains. Finally, cerulenin, a natural compound extensively studied for its antifungal potential through the inhibition of fatty acid biosynthesis, presented a lack of activity against most of the phytopathogens that could be correlated to its limited activity to yeasts and dimorphic fungi [66,67].

Therefore, the results obtained with the forty tested compounds validated the optimized assay conditions for the four phytopathogens. The developed HTS platform can characterize the activity of a wide diversity of synthetic and natural antifungal compounds and identify the known and new sensitivities and resistances of these four relevant phytopathogens.

### 3.8. Antifungal Potential of Microbial Extract Libraries

A proof of concept (POC) piloting HTS approach was also used with a small subset of 2400 microbial natural product extracts from the MEDINA Library. Extracts at 2xWBE (Whole Broth Equivalent) in 20% DMSO were screened at a final 1:10 dilution to avoid any inhibitory effects derived from the DMSO concentrations against the four pathogens. The antifungal activity observed in the agar-based assays was determined by measuring the diameter and turbidity of the inhibition zones (mm) when diameters were larger than 5 mm. Following this 5 mm cut-off, we obtained screening hit-rates ranging from 0.50% for *C. acutatum,* 1.88% for *F. proliferatum*, and 1.92% for *B. cinerea* to 2.13% for *M. grisea*.

**Figure 7:**
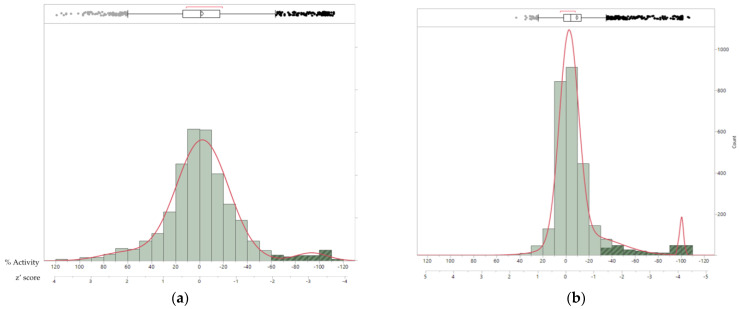
Population distribution histogram (bin size = 20%) of the antifungal activities of the 2400 microbial extracts tested against *B. cinerea*: (**a**) absorbance readouts and (**b**) fluorescence readouts. Distribution is represented according to the activity scale (% INH) and z’ score scale. Hit populations in the upper quartile of the boxplot are highlighted in dark green.

The antifungal activity was determined in microdilution assays as the percentage of inhibition (% INH), calculated for both the absorbance and fluorescence data. Figure 7a,b show a symmetrical and normal distribution around 0% INH of activity observed in the *B. cinerea* assay, indicating a low interference of the samples with both readouts. However, slight differences were observed between the activity distributions in the absorbance and fluorescence data. Statistical analysis showed that boxplots significatively highlighted the hit populations to the upper quartile, corresponding to a % INH > 60% for absorbance (Figure 7a) and a % INH > 35% for fluorescence (Figure 7b). Absorbance readouts provided greater dispersion in comparison to fluorescence due to the higher coefficient of variation of absorbance (CV = 14.87) compared to fluorescence (CV = 2.36). This could be explained by the fact that some extracts may promote phytopathogen growth, generating higher absorbance values, and this phytopathogen growth is minimized by the fluorescence of resazurin. Moreover, the fluorescence assay showed larger z’ scores that better separated the active samples from the general inactive population, also indicating more robust data. However, fluorescence readouts can be easily affected by certain sample components and/or their pH, which interferes with resazurin, generating false-positive signals. This effect can sometimes be challenging for the identification of active samples. Therefore, a hit selection criterion should only consider those extracts which are active in both assays. In this sense, only samples that inhibit fungal growth and also affect cell viability were selected. The implementation of these criteria resulted in hit rates of 0.71% for *C. acutatum,* 1.04% for *F. proliferatum,* 1.50% for *B. cinerea*, and 2.63% for *M. grisea*.

In general, we have shown that the developed protocols ensure a rapid and efficient HTS platform that allows for the profiling of the antifungal properties of a broad variety of samples (from synthetic to natural product compounds and extracts) using different assay methodologies (agar-based and liquid microdilution assays) for the high-quality selection of new potential inhibitors of the most relevant phytopathogens, namely *Botrytis cinerea*, *Colletotrichum acutatum*, *Fusarium proliferatum*, and *Magnaporthe grisea*. 

## 4. Conclusions

In this work, two HTS methodologies have been developed and validated to evaluate the antifungal effects of a large diversity of samples, pure compounds, and complex mixtures against four important fungal phytopathogens. The described approach can be applied for the development of new protocols for antifungal assays with additional strains. On the one hand, the previously developed agar-based assay allows us to fix some of the problems related to traditional agar tests, mainly regarding the time, the throughput of the assay, and the required sample volume. On the other hand, the microdilution method provides more information about the antifungal effects of samples as it can differentiate the inhibition of fungal growth (absorbance readouts) and cell viability (fluorescence readouts) within the same assay. Furthermore, this methodology is even more time-efficient, requires a lower quantity of fungal conidia, and has the potential to be scaled up to the 384-well format, allowing for the screening of a higher number of samples.

Both assay methods were validated using a large selection of synthetic and natural antifungal compounds covering a broad variety of modes of action. The MIC and IC_50_ values reported in the literature were in agreement with the results obtained using both assay set-ups (agar-based and microdilution assays), detecting activities at very low concentrations (<0.04 µg/mL). In addition and as previously reported, some differences were observed in the activities from agar and microdilution methods, revealing the importance of using both methodologies in the characterization of new antifungal agents. In general, both assay set-ups can identify hits according to their potency, which may result in more activity in one set-up than in the other due to the differential diffusion between the samples on the agar set-up, differential fungal growth rates, fungal physiology in the assay conditions, and/or specificity on their mechanisms of action. Therefore, future screening strategies to identify new fungicides should include both methodologies in order to collect more reliable data regarding the antifungal potential of sample libraries.

Finally, the POC piloting screening of a subset of 2400 natural product extracts validated the use of this HTS assay platform for the evaluation of sample libraries to identify new antifungal agents. Future studies will focus on the characterization of the chemical diversity of new bioactive metabolites detected in this preliminary screening and extending the use of the HTS phytopathogen platform for the discovery of new biocontrol products in plant protection.

## Figures and Tables

**Table 1:** Conidia concentrations obtained from the cultivation of the four phytopathogens in the 10 studied culture conditions. The most productive conditions for each fungal strain are highlighted in bold. Average and standard deviations were calculated with triplicate data from different batches.

Culture Media	Concentration (conidia/mL)			
	*B. cinerea*	*C. acutatum*	*F. proliferatum*	*M. grisea*
Solid Culture Media				
CMA	-	-	2.4 × 10^7^ ± 1.3	**2.7 × 10^8^ ± 1.0**
MEA	5.9 × 10^5^ ± 1.9	1.3 × 10^6^ ± 0.9	1.2 × 10^8^ ± 1.0	9.3 × 10^7^ ± 1.2
OAT	**3.7 × 10^7^ ± 1.5**	4.6 × 10^7^ ± 3.4	1.9 × 10^7^ ± 0.6	-
PDA	6.7 × 10^5^ ± 2.9	3.3 × 10^7^ ± 1.7	1.3 × 10^8^ ± 0.5	1.3 × 10^8^ ± 0.8
YM	8.3 × 10^5^ ± 2.9	**8.8 × 10^7^ ± 1.7**	**1.4 × 10^8^ ± 0.8**	1.9 × 10^8^ ± 0.9
Liquid Culture Media				
SDB	-	5.3 × 10^6^ ± 3.3	9.0 × 10^7^ ± 1.4	9.3 × 10^7^ ± 2.3
SMY	-	**7.8 × 10^7^ ± 1.9**	**2.5 × 10^8^ ± 0.4**	1.9 × 10^8^ ± 1.1
PDB	-	2.0 × 10^6^ ± 0.4	8.7 × 10^7^ ± 1.0	9.7 × 10^7^ ± 2.4
GMS	-	8.7 × 10^6^ ± 2.1	8.7 × 10^7^ ± 1.3	1.5 × 10^8^ ± 0.6
VME	-	2.9 × 10^7^ ± 1.3	1.4 × 10^8^ ± 0.5	**2.3 × 10^8^ ± 0.9**

(-) No conidia obtained after filtration, only mycelium development observed.

**Table 2:** Comparison of requirements to obtain a stock solution of fungal conidia from solid and liquid culture conditions.

Parameters	Solid Medium	Liquid Medium
Time of incubation	10–21 days	24–72 h
Volume of inoculum	200 mL (five Petri plates)	16 mL (one test tube)
Time to obtain conidia solution	1 h (Scraping + Filtration)	30 min (Filtration)

**Table 3:** Quality control parameters obtained for three different *B. cinerea* conidia concentrations for absorbance and fluorescence readouts. The condition that provided the most robust data is highlighted in bold.

Concentration of Inoculum	Absorbance		Fluorescence	
	S/B	RZ’	S/B	RZ’
**5 × 10^5^ conidia/mL**	15.31	0.76	11.74	0.76
**1 × 10^6^ conidia/mL**	**37.40**	**0.77**	**23.19**	**0.90**
**5 × 10^6^ conidia/mL**	2.33	<0	7.65	0.55

**Table 4:** Optimized conditions for the microdilution assays against the four phytopathogens.

Parameters	*Botrytis cinerea* B05.10	*Colletotrichum acutatum* CF-137177	*Fusarium proliferatum* CBS 115.97	*Magnaporthe grisea* CF-105765
Sporulation condition	OAT plates	SMY tube	SMY tube	VME tube
Time for sporulation	14–28 days	3 days	2 days	3 days
Assay medium	RPMI-1640	RPMI-1640	RPMI-1640	RPMI-1640
Concentration of conidia	1 × 10^6^ conidia/mL	1 × 10^6^ conidia/mL	1 × 10^6^ conidia/mL	1 × 10^6^ conidia/mL
Tolerance to DMSO	2%	2%	4%	2%
Growth inhibition assay (absorbance readout)				
Incubation time	72 h	40 h	20 h	48 h
RZ’ (*n* = 240 wells)	0.80 ± 0.05	0.75 ± 0.01	0.77 ± 0.05	0.81 ± 0.03
S/B (*n* = 240 wells)	48.95 ± 10.10	53.05 ± 5.50	57.71 ± 7.04	54.21 ± 5.89
IC_50_ AmB (µg/mL)	0.45 (0.33–0.60)	1.25 (1.12–1.33)	6.77 (6.11–7.51)	4.51 (4.33–4.69)
Viability inhibition assay (fluorescence readout)				
Incubation time (resazurin)	5–15 min	3 h	2 h	1 h 30 min
RZ’ (*n* = 240 wells)	0.94 ± 0.03	0.91 ± 0.01	0.94 ± 0.01	0.95 ± 0.01
S/B (*n* = 240 wells)	24.98 ± 4.90	17.01 ± 1.35	17.54 ± 1.76	13.62 ± 1.65
IC_50_ of AmB (µg/mL)	0.27 (0.25–0.30)	1.21 (1.19–1.23)	6.75 (6.41–7.12)	5.38 (5.30–5.46)

**Table 5:** In vitro activity of 40 commercial antifungal agents against *B. cinerea*, *C. acutatum*, *F. proliferatum*, and *M. grisea*. MIC values (µg/mL) correspond with the minimal concentration that inhibits fungal growth in the agar-based assay, and IC_50_ values (µg/mL) indicate the necessary concentration to inhibit 50% of conidia germination (Abs; absorbance readout) and cell viability (Flu; fluorescence readout); both parameters were obtained based on their DRC. The values in parenthesis correspond to confidence intervals of 95%. No activity detected at the maximal tested concentration is indicated as ‘*i*’ for *‘inactive’*.

Chemical Class	Compound	Mode of Action/ Target Site	*Botrytis cinerea*			*Colletotrichum acutatum*			*Fusarium proliferatum*			*Magnaporthe grisea*		
			Agar	Abs	Flu	Agar	Abs	Flu	Agar	Abs	Flu	Agar	Abs	Flu
			MIC	IC_50_	IC_50_	MIC	IC_50_	IC_50_	MIC	IC_50_	IC_50_	MIC	IC_50_	IC_50_
Acylalanines	benalaxyl	nucleic acids metabolism/RNA polymerase I	*i*	*i*	*i*	*i*	*i*	*i*	*i*	*i*	*i*	*i*	*i*	*i*
	metalaxyl		*i*	*i*	*i*	*i*	*i*	*i*	*i*	*i*	*i*	*i*	*i*	*i*
Anilino- pyrimidines	cyprodinil	protein synthesis	7.81	0.06 (0.05–0.07)	0.29 (0.24–0.35)	15.63	>20	14.99 (11.69–19.20)	*i*	8.28 (7.12–9.62)	14.33 (13.88–15.35)	*i*	16.02 (13.11–19.57)	>20
Benzimidazoles	benomyl	cytoskeleton and motor proteins/tubulin	62.5	0.06 (0.05–0.07)	0.06 (0.05–0.06)	125	0.35 (0.33–0.38)	0.46 (0.43–0.49)	500	0.98 (0.88–1.09)	0.95 (0.88–1.03)	1000	1.64 (1.51–1.78)	1.33 (1.29–1.37)
	thiabendazole		250	0.24 (0.21–0.28)	0.25 (0.21–0.30)	62.5	10.71 (9.66–11.87)	>20	1000	2.15 (1.95–2.36)	1.99 (1.84–2.15)	-	18.57 (17.86–19.31)	16.39 (16.04–16.75)
Cinnamic acid amides	dimethomorph	cell wall biosynthesis/ cellulose synthase	*i*	*i*	*i*	*i*	*i*	*i*	*i*	*i*	*i*	*i*	*i*	*i*
Cyano-methylene thiazolidines	flutianil	unknown	*i*	*i*	*i*	*i*	*i*	*i*	*i*	*i*	*i*	*i*	*i*	*i*
Cyclic nonribosomal peptides	cyclosporin A	signal transduction	125	1.27 (0.92–1.74)	2.20 (1.66–2.93	*i*	3.87 (3.198–4.70)	10.84 (10.27–11.44)	*i*	*i*	*i*	*i*	*i*	*i*
Dicarboximide	cycloheximide	protein synthesis	250	8.96 (7.27–11.05)	4.91 (4.05–5.96)	250	0.57 (0.49–0.66)	1.67 (1.53–1.82)	125	1.29 (1.07–1.56)	1.21 (1.11–1.31)	250	7.94 (7.44–8.48)	5.07 (4.90–5.25)
Dithio-carbamates	ferbam	multi-site contact	250	0.30 (0.28–0.32)	0.37 (0.36–0.38)	62.5	0.24 (0.22–0.25)	0.66 (0.63–0.70)	125	1.23 (1.08–1.41)	1.84 (1.75–1.94)	*i*	0.45 (0.43–0.47)	0.28 (0.28–0.29)
Ethylamino- thiazolecarboxamide	ethaboxam	cytoskeleton and motor proteins/tubulin	*i*	*i*	*i*	*i*	*i*	*i*	*i*	*i*	*i*	*i*	*i*	*i*
Glucopyranosyl antibiotics	validamycin	unknown/trehalase	*i*	*i*	*i*	*i*	*i*	*i*	*i*	*i*	*i*	*i*	*i*	*i*
Imidazolone	triflumizole	sterol biosynthesis/ C14-demethylase	62.5	<0.04	<0.04	15.63	<0.04	<0.04	7.81	0.26 (0.22–0.32)	0.28 (0.23–0.35)	31.25	0.05 (0.04–0.06)	<0.04
	fenamidone	respiration/complex III: *cyt bc1* at Qo site	*i*	*i*	*i*	500	11.33 (10.25–12.51)	>20	*i*	*i*	*i*	500	9.45 (8.79–10.16)	1.94 (1.85–2.02)
Macrocyclic	radicicol	histidine kinase/*Hsp90*	*i*	*i*	*i*	*i*	*i*	*i*	*i*	*i*	*i*	*i*	*i*	*i*
Mandelic acid amides	mandipropamid	cell wall biosynthesis/ cellulose synthase	*i*	*i*	*i*	*i*	*i*	*i*	*i*	*i*	*i*	*i*	*i*	*i*
Monocarboxylic acid amide	cerulenin	fatty acid biosynthesis/ b-ketoacyl-acyl carrier protein synthase	*i*	*i*	*i*	*i*	6.70 (5.64–7.96)	11.98 (11.47–12.51)	*i*	*i*	*i*	*i*	*i*	*i*
Mycotoxin	cytochalasin B	cell division	*i*	*i*	*i*	*i*	*i*	*i*	*i*	*i*	*i*	*i*	*i*	*i*
N-methoxy- (phenylethyl)- pyrazolecarboxamides	pydiflumetofen	respiration/complex II: succinate-dehydrogenase	15.63	0.07 (0.07–0.08)	0.12 (0.11–0.13)	*i*	6.32 (5.32–7.50)	2.08 (1.89–2.29)	15.63	<0.04	<0.04	15.62	0.14 (0.11–0.18)	<0.04
Nucleoside	tubercidin	protein synthesis	*i*	*i*	*i*	*i*	12.25 (9.69–15.49)	>20	*i*	*i*	*i*	*i*	*i*	*i*
	tunicamycin		125	2.62 (1.79–3.84)	2.55 (2.34–2.77)	500	4.05 (3.55–4.63)	6.71 (5.26–7.20)	*i*	*i*	*i*	*i*	*i*	*i*
Phenyl-oxo- ethylthiophene amide	isofetamid	respiration/complex II: succinate-dehydrogenase	31.25	0.16 (0.13–0.19)	0.40 (0.34–0.45)	*i*	*i*	*i*	*i*	14.00 (10.95–17.91)	19.94 (16.92–23.50)	62.5	1.49 (1.26–1.77)	0.23 (0.21–0.26)
Phenylpyrroles	fludioxonil	signal transduction/ MAP/Histidine Kinase	62.5	<0.04	0.07 (0.07–0.07)	31.25	0.07 (0.07–0.07)	0.07 (0.07–0.07)	31.25	0.09 (0.09–0.11)	<0.04	125	0.22 (0.21–0.24)	0.09 (0.09–0.09)
Picolinamides	fenpicoxamid	respiration/complex III: *cyt bc1* at Qi site	62.5	0.07 (0.06–0.08)	0.43 (0.32–0.57)	15.63	*i*	*i*	*i*	*i*	*i*	62.5	2.04 (1.61–2.59)	0.40 (0.39–0.42)
Pyridinecarboxamides	boscalid	respiration/complex II: succinate-dehydrogenase	125	0.33 (0.30–0.36)	0.70 (0.65–0.74)	*i*	*i*	*i*	*i*	*i*	*i*	*i*	*i*	*i*
Pyridinyl- ethylbenzamides	fluopyram		125	0.28 (0.25–0.32)	1.27 (1.17–1.37)	*i*	*i*	*i*	*i*	5.77 (4.08–8.15)	12.24 (10.49–14.28)	*i*	>20	17.28 (16.02–18.64)
Strobirulin	azoxystrobin	respiration/complex III: *cyt bc1* at Qo site	250	0.06 (0.06–0.07)	0.06 (0.05–0.07)	62.5	0.05 (0.04–0.05)	0.09 (0.09–0.09)	31.25	0.39 (0.32–0.49)	0.22 (0.19–0.26)	62.5	0.10 (0.09–0.11)	0.08 (0.07–0.08)
	mandestrobin		250	<0.04	0.78 (0.69–0.87)	250	0.12 (0.11–0.13)	0.08 (0.08–0.09)	31.25	0.12 (0.11–0.13)	0.04 (0.04–0.05)	500	2.27 (2.07–2.50)	0.82 (0.79–0.85)
	pyraclostrobin		31.25	<0.04	0.07 (0.05–0.09)	3.91	<0.04	<0.04	7.81	0.06 (0.05–0.07)	<0.04	1.95	<0.04	<0.04
	trifloxystrobin		31.25	0.88 (0.51–1.50)	1.99 (1.66–2.40)	7.81	6.41 (5.83–7.05)	6.43 (6.06–6.81)	7.81	7.23 (6.44–8.12)	7.79 (6.59–9.21)	3.90	1.83 (1.52–2.20)	1.59 (1.49–1.70)
	cyproconazole	sterol biosynthesis/ C14-demethylase	500	0.34 (0.27–0.43)	0.39 (0.35–0.44)	62.5	0.68 (0.61–0.76)	2.08 (1.93–2.22)	*i*	1.76 (1.17–2.64)	1.58 (1.37–1.81)	*i*	*i*	*i*
Triazoles	difenoconazole		250	0.44 (0.39–0.49)	0.81 (0.67–0.97)	250	0.16 (0.14–0.17)	0.61 (0.56–0.67)	125	0.70 (0.52–0.96)	0.38 (0.32–0.45)	1000	11.89 (11.34–12.47)	7.78 (7.34–8.25)
	fenbuconazole		500	0.20 (0.18–0.22)	0.43 (0.38–0.49)	*i*	0.23 (0.20–0.27)	0.58 (0.52–0.64)	*i*	1.82 (1.41–2.37)	1.39 (1.15–1.67)	*i*	*i*	*i*
	itraconazole		62.5	0.31 (0.19–0.51)	0.31 (0.29–0.33)	62.5	0.12 (0.11–0.13)	0.63 (0.58–0.70)	62.5	0.53 (0.41–0.68)	0.74 (0.62–0.87)	*i*	*i*	*i*
	mefentrifluconazole		250	0.07 (0.06–0.08)	0.11 (0.10–0.12)	62.5	0.11 (0.09–0.12)	0.28 (0.23–0.34)	1000	0.55 (0.42–0.71)	0.22 (0.20–0.25)	1000	*i*	*i*
	metconazole		250	0.06 (0.05–0.05)	0.10 (0.09–0.11)	62.5	<0.04	0.05 (0.05–0.06)	31.25	<0.04	<0.04	500	4.15 (3.86–4.47)	4.16 (3.91–4.41)
	tetraconazole		*i*	12.52 (7.73–20.29)	5.40 (4.96–5.88)	*i*	1.30 (0.97–1.75)	3.16 (2.89–3.46)	*i*	8.02 (6.26–10.29)	9.07 (8.29–9.92)	*i*	*i*	*i*
	flutriafol		*i*	11.02 (9.69–12.55)	>20	*i*	6.82 (6.26–7.43)	6.48 (6.16–6.82)	*i*	3.19 (2.79–3.64)	3.38 (3.09–3.70)	*i*	*i*	*i*
Valinamide carbamates	iprovalicarb	cell wall biosynthesis/ cellulose synthase	*i*	*i*	*i*	*i*	*i*	*i*	*i*	*i*	*i*	*i*	*i*	*i*

## Data Availability

Not applicable.
